# Diaqua­[(1*R*,2*S*,4*R*,8*R*,9*S*,11*R*)-2,9-di­methyl-1,4,8,11-tetra­aza­cyclo­tetra­decane]nickel(II) dichloride dihydrate

**DOI:** 10.1107/S160053681203276X

**Published:** 2012-07-25

**Authors:** James Alan Townsend, John Desper

**Affiliations:** a Kansas Wesleyan University, 100 East Claflin, Salina, Kansas 67401, USA; b Kansas State University, Manhattan, Kansas 66502, USA

## Abstract

The crystal structure of the title complex, [Ni(C_12_H_28_N_4_)(H_2_O)_2_]Cl_2_·2H_2_O, displays O—H⋯Cl and O—H⋯O hydrogen bonding. The tetra­aza­cyclo­tetra­decane ligand inter­acts with the Ni^II^ atom in the *cis* V configuration and the final two ligand binding sites are occupied by water.

## Related literature

For uses of the title compound, see: Kimura *et al.* (1992 ▶); Liang *et al.* (2002 ▶); Burrows *et al.* (1992 ▶, 1988 ▶); Kelly *et al.* (1999 ▶); Churchard *et al.* (2010 ▶). For the synthesis of the ligand, see: Beck & Lang (2003 ▶); Beck *et al.* (1998 ▶, 2003 ▶). For metal complex formation, see: Sadler *et al.* (2007 ▶); Voelcker *et al.* (2008 ▶). For nickel cyclam complex crystal structures with a *cis*-V configuration, see: Sadler *et al.* (2007 ▶); Ito *et al.* (1981 ▶, 1982 ▶); Allen (2002 ▶). For details of peptide racemization, see: Liardon & Ledermann (1986 ▶).
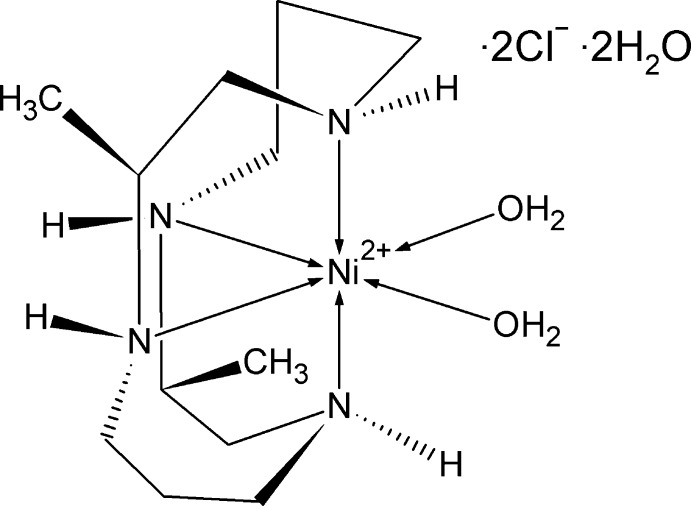


## Experimental

### 

#### Crystal data

[Ni(C_12_H_28_N_4_)(H_2_O)_2_]Cl_2_·2H_2_O
*M* *_r_* = 430.06
Orthorhombic, 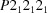

*a* = 9.7309 (8) Å
*b* = 14.0994 (11) Å
*c* = 14.6000 (11) Å
*V* = 2003.1 (3) Å^3^
*Z* = 4
Mo *K*α radiation
μ = 1.26 mm^−1^
*T* = 120 K
0.28 × 0.24 × 0.12 mm

#### Data collection

Bruker APEXII CCD diffractometer
Absorption correction: multi-scan (*SADABS*; Bruker, 2009 ▶) *T*_min_ = 0.720, *T*_max_ = 0.864
25180 measured reflections
7454 independent reflections
6563 reflections with *I* > σ(*I*)
*R*_int_ = 0.061

#### Refinement

*R*[*F* ^2^ > 2σ(*F* ^2^)] = 0.033
*wR*(*F* ^2^) = 0.083
*S* = 1.04
7454 reflections
246 parameters
H atoms treated by a mixture of independent and constrained refinement
Δρ_max_ = 0.29 e Å^−3^
Δρ_min_ = −0.38 e Å^−3^
Absolute structure: Flack (1983 ▶), 3205 Friedel pairs
Flack parameter: −0.006 (7)

### 

Data collection: *APEX2* (Bruker, 2009 ▶); cell refinement: *SAINT* (Bruker, 2009 ▶); data reduction: *SAINT*; program(s) used to solve structure: *SHELXS97* (Sheldrick, 2008 ▶); program(s) used to refine structure: *SHELXL97* (Sheldrick, 2008 ▶); molecular graphics: *XP* in *SHELXTL* (Sheldrick, 2008 ▶); software used to prepare material for publication: *XCIF* in *SHELXTL*.

## Supplementary Material

Crystal structure: contains datablock(s) I, global. DOI: 10.1107/S160053681203276X/pk2429sup1.cif

Supplementary material file. DOI: 10.1107/S160053681203276X/pk2429Isup2.mol

Structure factors: contains datablock(s) I. DOI: 10.1107/S160053681203276X/pk2429Isup3.hkl

Additional supplementary materials:  crystallographic information; 3D view; checkCIF report

## Figures and Tables

**Table 1:** Hydrogen-bond geometry (Å, °)

*D*—H⋯*A*	*D*—H	H⋯*A*	*D*⋯*A*	*D*—H⋯*A*
O1—H1*A*⋯Cl1	0.73 (2)	2.44 (2)	3.1580 (12)	172 (2)
O1—H1*B*⋯O3	0.85 (2)	1.84 (2)	2.6912 (17)	176 (2)
O2—H2*A*⋯Cl2	0.74 (2)	2.38 (2)	3.1192 (13)	176 (2)
O2—H2*B*⋯O4	0.80 (2)	1.88 (2)	2.6767 (18)	177 (2)
O3—H3*A*⋯Cl2	0.81 (2)	2.50 (3)	3.2587 (15)	156 (2)
O3—H3*B*⋯Cl1^i^	0.78 (2)	2.48 (2)	3.2256 (14)	160 (2)
O4—H4*A*⋯Cl1	0.75 (3)	2.50 (3)	3.1971 (18)	154 (2)
O4—H4*B*⋯Cl1^ii^	0.72 (3)	2.50 (3)	3.2227 (16)	173 (3)

Symmetry codes: (i) 
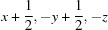
; (ii) 
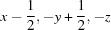
.

**Fig. 1.**
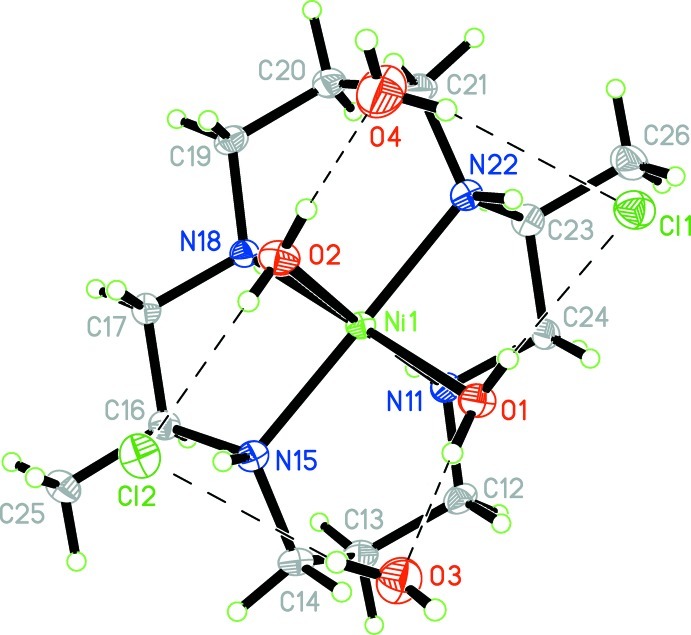
The molecular structure of the title compound, showing 50% probability displacement ellipsoids.

## supplementary crystallographic information

### Comment

The ligand in the title compound was synthesized by base-catalyzed metal-templated cyclization of dipeptides, metal removal using HCl and finally amide reduction to yield a C-functionalized cyclam molecule (Beck & Lang, 2003). The stereochemical integrity of the ligands previously synthesized, however, was never established. Strong bases such as NaOMe, which is used in the ligand synthesis, have the ability to racemize peptides Liardon *et al.* (1986). The crystal structure of the title compound shows that the stereochemical integrity of the (2*S*,9*S*)-2,9-dimethyl-1,4,8,11-tetraazacyclotetradecane ligand is maintained throughout the synthesis.

Cyclam metal complexes have 6 possible configurations: *trans* I—V and *cis* V (Liang *et al.*, 2002). Typically the structure of cyclam metal complexes tends to favor the thermodynamically most stable *trans* III configuration in the solid state (Liang *et al.*, 2002). However, in this case the compound has adopted the *cis* V configuration with two water molecules acting as ligands to the metal center. The chloride counter ions interact with the water ligands through O—H···Cl hydrogen bonds. Similarly configured nickel cyclam complexes were reported by Ito *et al.* (1981, 1982). A recent crystal structure search in the CCDC database has shown that only 4% of cyclam complexes without nitrogen functionalization, utilizing halogen containing counter ions and monodentate ligands for the final two coordination sites, adopt a *cis* V configuration (Allen, 2002).

When chiral carbons are present in the cyclam it is possible to generate two stereoisomers for each metal complex configuration. The diastereomers for each configuration are dependent on the chirality around the N atoms in the complex as the carbon chirality in the cyclam ligand is both encoded and maintained during synthesis. The title compound has adopted a diastereomer that places the methyl side arms into the equatorial plane of the 5 membered rings. This minimizes steric interactions with the remainder of the cyclam and the water ligands attached to the nickel center.

### Experimental

The (2*S*,9*S*)-2,9-dimethyl-1,4,8,11-tetraazacyclotetradecane (100 mg, 0.44 mmol) was dissolved in methanol (2 ml) and the NiCl_2._6H_2_O (105 mg, 0.44 mmol) was added in methanol (2 ml). The reaction mixture was heated to reflux for ten minutes and allowed to cool. A purple crystalline solid was isolated for X-ray analysis after 4 months of crystallization *via* slow evaporation at RT.

### Refinement

All hydrogen atoms, excepting amine and water H atoms, were placed in idealized positions and allowed to ride. Coordinates of the amine and water H atoms were allowed to refine. Absolute configuration was determined by inspection of the Flack parameter produced by least-squares refinement. A value of -0.006 (7) indicated that the chosen configuration was correct.

### Crystal data

**Table:**

[Ni(C_12_H_28_N_4_)(H_2_O)_2_]Cl_2_·2H_2_O	*F*(000) = 920
*M**_r_* = 430.06	*D*_x_ = 1.426 Mg m^−^^3^
Orthorhombic, *P*2_1_2_1_2_1_	Mo *K*α radiation, λ = 0.71073 Å
Hall symbol: P 2ac 2ab	Cell parameters from 9955 reflections
*a* = 9.7309 (8) Å	θ = 3.1–33.2°
*b* = 14.0994 (11) Å	µ = 1.26 mm^−^^1^
*c* = 14.6000 (11) Å	*T* = 120 K
*V* = 2003.1 (3) Å^3^	Plate, purple
*Z* = 4	0.28 × 0.24 × 0.12 mm

### Data collection

**Table:**

Bruker APEXII CCD diffractometer	7454 independent reflections
Radiation source: fine-focus sealed tube	6563 reflections with *I* > σ(*I*)
Graphite monochromator	*R*_int_ = 0.061
φ and ω scans	θ_max_ = 33.1°, θ_min_ = 2.9°
Absorption correction: multi-scan (*SADABS*; Bruker, 2009)	*h* = −14→14
*T*_min_ = 0.720, *T*_max_ = 0.864	*k* = −20→21
25180 measured reflections	*l* = −22→21

### Refinement

**Table:**

Refinement on *F*^2^	Secondary atom site location: difference Fourier map
Least-squares matrix: full	Hydrogen site location: inferred from neighbouring sites
*R*[*F*^2^ > 2σ(*F*^2^)] = 0.033	H atoms treated by a mixture of independent and constrained refinement
*wR*(*F*^2^) = 0.083	*w* = 1/[σ^2^(*F*_o_^2^) + (0.035*P*)^2^] where *P* = (*F*_o_^2^ + 2*F*_c_^2^)/3
*S* = 1.04	(Δ/σ)_max_ = 0.001
7454 reflections	Δρ_max_ = 0.29 e Å^−^^3^
246 parameters	Δρ_min_ = −0.38 e Å^−^^3^
0 restraints	Absolute structure: Flack (1983), 3205 Friedel pairs
Primary atom site location: structure-invariant direct methods	Flack parameter: −0.006 (7)

### Special details

**Table:**

Geometry. All e.s.d.'s (except the e.s.d. in the dihedral angle between two l.s. planes) are estimated using the full covariance matrix. The cell e.s.d.'s are taken into account individually in the estimation of e.s.d.'s in distances, angles and torsion angles; correlations between e.s.d.'s in cell parameters are only used when they are defined by crystal symmetry. An approximate (isotropic) treatment of cell e.s.d.'s is used for estimating e.s.d.'s involving l.s. planes.
Refinement. Refinement of *F*^2^ against ALL reflections. The weighted *R*-factor *wR* and goodness of fit *S* are based on *F*^2^, conventional *R*-factors *R* are based on *F*, with *F* set to zero for negative *F*^2^. The threshold expression of *F*^2^ > σ(*F*^2^) is used only for calculating *R*-factors(gt) *etc*. and is not relevant to the choice of reflections for refinement. *R*-factors based on *F*^2^ are statistically about twice as large as those based on *F*, and *R*- factors based on ALL data will be even larger.

### Fractional atomic coordinates and isotropic or equivalent isotropic displacement parameters (Å^2^)

**Table:**

	*x*	*y*	*z*	*U*_iso_*/*U*_eq_	
Cl1	0.71927 (4)	0.16329 (3)	0.06960 (2)	0.02112 (8)	
Cl2	0.79391 (5)	0.60262 (3)	0.074484 (19)	0.02032 (8)	
Ni1	0.749450 (19)	0.382317 (11)	0.280156 (10)	0.01238 (5)	
O1	0.87944 (13)	0.32002 (8)	0.18131 (7)	0.0182 (2)	
H1A	0.841 (2)	0.2877 (15)	0.1520 (12)	0.022*	
H1B	0.925 (2)	0.3587 (13)	0.1483 (12)	0.022*	
O2	0.63308 (14)	0.43853 (8)	0.16958 (6)	0.0188 (2)	
H2A	0.670 (3)	0.4766 (14)	0.1447 (12)	0.023*	
H2B	0.590 (2)	0.4070 (14)	0.1346 (13)	0.023*	
O3	1.03357 (16)	0.44143 (10)	0.08293 (8)	0.0264 (3)	
H3A	0.985 (3)	0.4846 (15)	0.0655 (13)	0.032*	
H3B	1.069 (3)	0.4260 (15)	0.0375 (15)	0.032*	
O4	0.49336 (18)	0.32714 (12)	0.05395 (10)	0.0389 (4)	
H4A	0.538 (3)	0.2856 (19)	0.0410 (16)	0.047*	
H4B	0.436 (3)	0.3292 (19)	0.0224 (16)	0.047*	
N11	0.87279 (15)	0.31679 (9)	0.37995 (8)	0.0157 (2)	
H11	0.838 (2)	0.3325 (13)	0.4315 (10)	0.019*	
C12	1.02074 (17)	0.33903 (11)	0.37783 (10)	0.0191 (3)	
H12A	1.0595	0.3182	0.3185	0.023*	
H12B	1.0678	0.3032	0.4270	0.023*	
C13	1.04857 (18)	0.44409 (11)	0.39043 (10)	0.0200 (3)	
H13A	1.1473	0.4528	0.4045	0.024*	
H13B	0.9956	0.4669	0.4440	0.024*	
C14	1.01209 (17)	0.50543 (11)	0.30776 (9)	0.0181 (3)	
H14A	1.0482	0.5702	0.3178	0.022*	
H14B	1.0581	0.4792	0.2528	0.022*	
N15	0.86136 (14)	0.51134 (9)	0.28990 (7)	0.0151 (2)	
H15	0.849 (2)	0.5386 (13)	0.2377 (11)	0.018*	
C16	0.79010 (17)	0.57511 (10)	0.35683 (9)	0.0153 (3)	
H16A	0.8226	0.5585	0.4198	0.018*	
C17	0.63766 (18)	0.55513 (10)	0.35165 (9)	0.0168 (3)	
H17A	0.5890	0.5921	0.3993	0.020*	
H17B	0.6020	0.5749	0.2911	0.020*	
N18	0.61136 (14)	0.45273 (8)	0.36543 (7)	0.0143 (2)	
H18	0.634 (2)	0.4368 (13)	0.4203 (11)	0.017*	
C19	0.46435 (17)	0.43027 (11)	0.35336 (10)	0.0186 (3)	
H19A	0.4358	0.4481	0.2906	0.022*	
H19B	0.4096	0.4684	0.3971	0.022*	
C20	0.43414 (18)	0.32571 (11)	0.36862 (10)	0.0192 (3)	
H20A	0.3335	0.3175	0.3747	0.023*	
H20B	0.4765	0.3060	0.4273	0.023*	
C21	0.48560 (17)	0.26014 (11)	0.29316 (10)	0.0187 (3)	
H21A	0.4471	0.1960	0.3034	0.022*	
H21B	0.4504	0.2833	0.2336	0.022*	
N22	0.63722 (14)	0.25275 (9)	0.28777 (7)	0.0149 (2)	
H22	0.662 (2)	0.2237 (13)	0.2382 (11)	0.018*	
C23	0.69697 (18)	0.19430 (10)	0.36349 (9)	0.0174 (3)	
H23A	0.6546	0.2156	0.4224	0.021*	
C24	0.84928 (18)	0.21402 (10)	0.36830 (9)	0.0170 (3)	
H24A	0.8900	0.1791	0.4205	0.020*	
H24B	0.8943	0.1919	0.3114	0.020*	
C25	0.8201 (2)	0.67969 (11)	0.34008 (10)	0.0228 (3)	
H25A	0.9188	0.6913	0.3471	0.034*	
H25B	0.7692	0.7182	0.3845	0.034*	
H25C	0.7916	0.6968	0.2779	0.034*	
C26	0.6686 (2)	0.08823 (11)	0.35262 (11)	0.0266 (4)	
H26A	0.5693	0.0770	0.3544	0.040*	
H26B	0.7130	0.0534	0.4027	0.040*	
H26C	0.7055	0.0663	0.2939	0.040*	

### Atomic displacement parameters (Å^2^)

**Table:**

	*U*^11^	*U*^22^	*U*^33^	*U*^12^	*U*^13^	*U*^23^
Cl1	0.0206 (2)	0.02300 (17)	0.01969 (14)	−0.00200 (14)	−0.00002 (13)	−0.00204 (11)
Cl2	0.0262 (2)	0.02052 (16)	0.01430 (13)	−0.00036 (15)	0.00089 (13)	0.00095 (11)
Ni1	0.01140 (10)	0.01451 (8)	0.01122 (7)	−0.00030 (8)	−0.00007 (7)	−0.00057 (5)
O1	0.0176 (6)	0.0210 (5)	0.0159 (4)	−0.0025 (5)	0.0020 (4)	−0.0034 (4)
O2	0.0185 (6)	0.0224 (5)	0.0155 (4)	−0.0030 (5)	−0.0025 (4)	0.0017 (4)
O3	0.0282 (8)	0.0289 (6)	0.0221 (5)	0.0022 (6)	0.0062 (5)	0.0027 (4)
O4	0.0328 (9)	0.0410 (8)	0.0428 (7)	0.0097 (7)	−0.0187 (6)	−0.0186 (6)
N11	0.0148 (6)	0.0176 (5)	0.0146 (4)	0.0005 (5)	−0.0009 (4)	−0.0020 (4)
C12	0.0134 (7)	0.0229 (7)	0.0210 (6)	0.0025 (6)	−0.0025 (5)	−0.0015 (5)
C13	0.0148 (8)	0.0225 (7)	0.0225 (6)	−0.0013 (6)	−0.0054 (6)	−0.0016 (5)
C14	0.0129 (7)	0.0220 (7)	0.0193 (6)	−0.0025 (6)	0.0014 (5)	−0.0002 (5)
N15	0.0149 (6)	0.0177 (5)	0.0128 (4)	0.0003 (5)	0.0005 (4)	−0.0009 (4)
C16	0.0150 (7)	0.0164 (6)	0.0145 (5)	−0.0011 (5)	0.0003 (5)	−0.0017 (4)
C17	0.0173 (8)	0.0145 (6)	0.0186 (5)	0.0008 (6)	0.0005 (5)	−0.0004 (5)
N18	0.0131 (6)	0.0147 (5)	0.0150 (5)	−0.0004 (5)	−0.0008 (4)	0.0002 (4)
C19	0.0126 (8)	0.0216 (7)	0.0215 (6)	0.0009 (6)	0.0014 (5)	−0.0012 (5)
C20	0.0151 (8)	0.0198 (7)	0.0228 (6)	−0.0015 (6)	0.0036 (6)	−0.0020 (5)
C21	0.0135 (7)	0.0214 (7)	0.0211 (6)	−0.0022 (6)	−0.0015 (5)	−0.0024 (5)
N22	0.0150 (6)	0.0178 (5)	0.0119 (4)	0.0002 (5)	−0.0005 (4)	−0.0009 (4)
C23	0.0191 (8)	0.0184 (6)	0.0146 (5)	−0.0002 (6)	−0.0006 (5)	0.0016 (4)
C24	0.0163 (8)	0.0151 (6)	0.0195 (6)	0.0001 (6)	−0.0019 (5)	0.0016 (5)
C25	0.0216 (9)	0.0175 (7)	0.0294 (7)	−0.0043 (6)	−0.0001 (6)	−0.0024 (5)
C26	0.0250 (10)	0.0188 (7)	0.0359 (8)	−0.0032 (7)	−0.0037 (7)	0.0062 (6)

### Geometric parameters (Å, º)

**Table:**

Ni1—N18	2.0836 (12)	C16—C25	1.523 (2)
Ni1—N11	2.1017 (13)	C16—H16A	1.0000
Ni1—O1	2.1105 (11)	C17—N18	1.4800 (19)
Ni1—N15	2.1249 (13)	C17—H17A	0.9900
Ni1—O2	2.1253 (11)	C17—H17B	0.9900
Ni1—N22	2.1312 (13)	N18—C19	1.476 (2)
O1—H1A	0.73 (2)	N18—H18	0.861 (16)
O1—H1B	0.85 (2)	C19—C20	1.520 (2)
O2—H2A	0.74 (2)	C19—H19A	0.9900
O2—H2B	0.80 (2)	C19—H19B	0.9900
O3—H3A	0.81 (2)	C20—C21	1.523 (2)
O3—H3B	0.78 (2)	C20—H20A	0.9900
O4—H4A	0.75 (3)	C20—H20B	0.9900
O4—H4B	0.72 (3)	C21—N22	1.481 (2)
N11—C12	1.474 (2)	C21—H21A	0.9900
N11—C24	1.4767 (19)	C21—H21B	0.9900
N11—H11	0.853 (15)	N22—C23	1.4965 (18)
C12—C13	1.517 (2)	N22—H22	0.864 (17)
C12—H12A	0.9900	C23—C24	1.510 (3)
C12—H12B	0.9900	C23—C26	1.529 (2)
C13—C14	1.527 (2)	C23—H23A	1.0000
C13—H13A	0.9900	C24—H24A	0.9900
C13—H13B	0.9900	C24—H24B	0.9900
C14—N15	1.492 (2)	C25—H25A	0.9800
C14—H14A	0.9900	C25—H25B	0.9800
C14—H14B	0.9900	C25—H25C	0.9800
N15—C16	1.4980 (18)	C26—H26A	0.9800
N15—H15	0.861 (17)	C26—H26B	0.9800
C16—C17	1.512 (2)	C26—H26C	0.9800
			
N18—Ni1—N11	99.41 (5)	N18—C17—H17A	109.6
N18—Ni1—O1	173.42 (4)	C16—C17—H17A	109.6
N11—Ni1—O1	87.06 (4)	N18—C17—H17B	109.6
N18—Ni1—N15	83.26 (5)	C16—C17—H17B	109.6
N11—Ni1—N15	92.14 (5)	H17A—C17—H17B	108.1
O1—Ni1—N15	95.45 (5)	C19—N18—C17	111.14 (12)
N18—Ni1—O2	86.14 (5)	C19—N18—Ni1	116.84 (9)
N11—Ni1—O2	174.18 (5)	C17—N18—Ni1	105.79 (9)
O1—Ni1—O2	87.43 (5)	C19—N18—H18	107.6 (14)
N15—Ni1—O2	90.27 (5)	C17—N18—H18	109.7 (13)
N18—Ni1—N22	92.68 (5)	Ni1—N18—H18	105.5 (13)
N11—Ni1—N22	83.09 (5)	N18—C19—C20	112.22 (13)
O1—Ni1—N22	89.20 (5)	N18—C19—H19A	109.2
N15—Ni1—N22	173.17 (5)	C20—C19—H19A	109.2
O2—Ni1—N22	94.95 (5)	N18—C19—H19B	109.2
Ni1—O1—H1A	110.9 (18)	C20—C19—H19B	109.2
Ni1—O1—H1B	115.6 (13)	H19A—C19—H19B	107.9
H1A—O1—H1B	109.4 (19)	C19—C20—C21	114.79 (12)
Ni1—O2—H2A	112.7 (18)	C19—C20—H20A	108.6
Ni1—O2—H2B	124.1 (14)	C21—C20—H20A	108.6
H2A—O2—H2B	110 (2)	C19—C20—H20B	108.6
H3A—O3—H3B	102 (2)	C21—C20—H20B	108.6
H4A—O4—H4B	108 (3)	H20A—C20—H20B	107.5
C12—N11—C24	110.96 (13)	N22—C21—C20	114.12 (13)
C12—N11—Ni1	116.73 (9)	N22—C21—H21A	108.7
C24—N11—Ni1	105.25 (9)	C20—C21—H21A	108.7
C12—N11—H11	110.4 (14)	N22—C21—H21B	108.7
C24—N11—H11	107.2 (13)	C20—C21—H21B	108.7
Ni1—N11—H11	105.8 (13)	H21A—C21—H21B	107.6
N11—C12—C13	112.31 (14)	C21—N22—C23	112.74 (12)
N11—C12—H12A	109.1	C21—N22—Ni1	116.93 (9)
C13—C12—H12A	109.1	C23—N22—Ni1	108.15 (9)
N11—C12—H12B	109.1	C21—N22—H22	110.6 (15)
C13—C12—H12B	109.1	C23—N22—H22	104.5 (12)
H12A—C12—H12B	107.9	Ni1—N22—H22	102.7 (13)
C12—C13—C14	114.56 (12)	N22—C23—C24	108.32 (12)
C12—C13—H13A	108.6	N22—C23—C26	113.08 (13)
C14—C13—H13A	108.6	C24—C23—C26	111.22 (14)
C12—C13—H13B	108.6	N22—C23—H23A	108.0
C14—C13—H13B	108.6	C24—C23—H23A	108.0
H13A—C13—H13B	107.6	C26—C23—H23A	108.0
N15—C14—C13	113.47 (13)	N11—C24—C23	109.77 (13)
N15—C14—H14A	108.9	N11—C24—H24A	109.7
C13—C14—H14A	108.9	C23—C24—H24A	109.7
N15—C14—H14B	108.9	N11—C24—H24B	109.7
C13—C14—H14B	108.9	C23—C24—H24B	109.7
H14A—C14—H14B	107.7	H24A—C24—H24B	108.2
C14—N15—C16	112.00 (11)	C16—C25—H25A	109.5
C14—N15—Ni1	117.88 (9)	C16—C25—H25B	109.5
C16—N15—Ni1	108.68 (9)	H25A—C25—H25B	109.5
C14—N15—H15	108.2 (15)	C16—C25—H25C	109.5
C16—N15—H15	104.2 (13)	H25A—C25—H25C	109.5
Ni1—N15—H15	104.7 (13)	H25B—C25—H25C	109.5
N15—C16—C17	108.04 (11)	C23—C26—H26A	109.5
N15—C16—C25	112.81 (12)	C23—C26—H26B	109.5
C17—C16—C25	111.14 (13)	H26A—C26—H26B	109.5
N15—C16—H16A	108.2	C23—C26—H26C	109.5
C17—C16—H16A	108.2	H26A—C26—H26C	109.5
C25—C16—H16A	108.2	H26B—C26—H26C	109.5
N18—C17—C16	110.16 (13)		
			
N18—Ni1—N11—C12	−122.05 (10)	N11—Ni1—N18—C19	−122.13 (10)
O1—Ni1—N11—C12	56.81 (10)	N15—Ni1—N18—C19	146.81 (10)
N15—Ni1—N11—C12	−38.54 (10)	O2—Ni1—N18—C19	56.08 (10)
N22—Ni1—N11—C12	146.36 (11)	N22—Ni1—N18—C19	−38.70 (10)
N18—Ni1—N11—C24	114.41 (10)	N11—Ni1—N18—C17	113.58 (9)
O1—Ni1—N11—C24	−66.72 (10)	N15—Ni1—N18—C17	22.53 (9)
N15—Ni1—N11—C24	−162.07 (10)	O2—Ni1—N18—C17	−68.20 (9)
N22—Ni1—N11—C24	22.83 (10)	N22—Ni1—N18—C17	−162.98 (9)
C24—N11—C12—C13	−179.52 (11)	C17—N18—C19—C20	−179.10 (11)
Ni1—N11—C12—C13	59.94 (14)	Ni1—N18—C19—C20	59.39 (13)
N11—C12—C13—C14	−72.87 (18)	N18—C19—C20—C21	−71.94 (17)
C12—C13—C14—N15	68.35 (18)	C19—C20—C21—N22	68.51 (18)
C13—C14—N15—C16	75.20 (15)	C20—C21—N22—C23	74.12 (15)
C13—C14—N15—Ni1	−52.00 (14)	C20—C21—N22—Ni1	−52.15 (14)
N18—Ni1—N15—C14	134.12 (9)	N18—Ni1—N22—C21	35.07 (10)
N11—Ni1—N15—C14	34.89 (9)	N11—Ni1—N22—C21	134.24 (10)
O1—Ni1—N15—C14	−52.36 (9)	O1—Ni1—N22—C21	−138.62 (10)
O2—Ni1—N15—C14	−139.80 (9)	O2—Ni1—N22—C21	−51.28 (10)
N18—Ni1—N15—C16	5.35 (9)	N18—Ni1—N22—C23	−93.44 (9)
N11—Ni1—N15—C16	−93.88 (9)	N11—Ni1—N22—C23	5.72 (9)
O1—Ni1—N15—C16	178.86 (9)	O1—Ni1—N22—C23	92.86 (9)
O2—Ni1—N15—C16	91.42 (9)	O2—Ni1—N22—C23	−179.79 (9)
C14—N15—C16—C17	−163.86 (11)	C21—N22—C23—C24	−163.87 (12)
Ni1—N15—C16—C17	−31.87 (12)	Ni1—N22—C23—C24	−33.01 (13)
C14—N15—C16—C25	72.87 (16)	C21—N22—C23—C26	72.38 (17)
Ni1—N15—C16—C25	−155.13 (11)	Ni1—N22—C23—C26	−156.76 (12)
N15—C16—C17—N18	54.08 (14)	C12—N11—C24—C23	−175.69 (11)
C25—C16—C17—N18	178.35 (10)	Ni1—N11—C24—C23	−48.56 (12)
C16—C17—N18—C19	−175.47 (11)	N22—C23—C24—N11	55.86 (14)
C16—C17—N18—Ni1	−47.70 (12)	C26—C23—C24—N11	−179.28 (11)

### Hydrogen-bond geometry (Å, º)

**Table:**

*D*—H···*A*	*D*—H	H···*A*	*D*···*A*	*D*—H···*A*
O1—H1*A*···Cl1	0.73 (2)	2.44 (2)	3.1580 (12)	172 (2)
O1—H1*B*···O3	0.85 (2)	1.84 (2)	2.6912 (17)	176 (2)
O2—H2*A*···Cl2	0.74 (2)	2.38 (2)	3.1192 (13)	176 (2)
O2—H2*B*···O4	0.80 (2)	1.88 (2)	2.6767 (18)	177 (2)
O3—H3*A*···Cl2	0.81 (2)	2.50 (3)	3.2587 (15)	156 (2)
O3—H3*B*···Cl1^i^	0.78 (2)	2.48 (2)	3.2256 (14)	160 (2)
O4—H4*A*···Cl1	0.75 (3)	2.50 (3)	3.1971 (18)	154 (2)
O4—H4*B*···Cl1^ii^	0.72 (3)	2.50 (3)	3.2227 (16)	173 (3)

Symmetry codes: (i) *x*+1/2, −*y*+1/2, −*z*; (ii) *x*−1/2, −*y*+1/2, −*z*.
